# E-Skin: The Dawn of a New Era of On-Body Monitoring Systems

**DOI:** 10.3390/mi12091091

**Published:** 2021-09-10

**Authors:** Alina-Cristina Bunea, Violeta Dediu, Edwin Alexandru Laszlo, Florian Pistriţu, Mihaela Carp, Florina Silvia Iliescu, Octavian Narcis Ionescu, Ciprian Iliescu

**Affiliations:** 1 National Institute for Research and Development in Microtechnologies—IMT, 077190 Bucharest, Romania; alina.bunea@imt.ro (A.-C.B.); violeta.dediu@imt.ro (V.D.); edwin.laszlo@imt.ro (E.A.L.); florian.pistritu@imt.ro (F.P.); mihaela.carp@imt.ro (M.C.); florina.iliescu@imt.ro (F.S.I.); octavian.ionescu@imt.ro (O.N.I.); 2 Faculty of Electrical and Mechanical Engineering, Petroleum-Gas University of Ploiesti, 100680 Ploiesti, Romania; 3 Academy of Romanian Scientists, 010071 Bucharest, Romania; 4 Faculty of Applied Chemistry and Materials Science, University “Politehnica” of Bucharest, 011061 Bucharest, Romania

**Keywords:** electronic skin (e-skin), flexible electronics, wearable devices, health monitoring sensors

## Abstract

Real-time “on-body” monitoring of human physiological signals through wearable systems developed on flexible substrates (e-skin) is the next target in human health control and prevention, while an alternative to bulky diagnostic devices routinely used in clinics. The present work summarizes the recent trends in the development of e-skin systems. Firstly, we revised the material development for e-skin systems. Secondly, aspects related to fabrication techniques were presented. Next, the main applications of e-skin systems in monitoring, such as temperature, pulse, and other bio-electric signals related to health status, were analyzed. Finally, aspects regarding the power supply and signal processing were discussed. The special features of e-skin as identified contribute clearly to the developing potential as in situ diagnostic tool for further implementation in clinical practice at patient personal levels.

## 1. Introduction

The skin, the largest human organ, weighs about 16% of the total body weight and completes numerous and various functions. First and foremost, it is a powerful physical and immunological barrier between the body and the external environment while providing efficient housing for the muscles, bones, internal organs, and fluids. The skin also acts as a highly efficient temperature controller and, to some extent, a hydro-electrolytic sensor, and regulator. Skin provides tactile, thermal, and pain-related information as the primary interface between the external environment and the central nervous system [1]. This remarkable multi-functional organ possesses excellent elasticity and healing abilities. Suppose we were to translate its biological functions into electronics terminology. In this case, the skin is a massive array of highly sensitive sensors with negative feedback control loops, able to process a considerable amount of data in real-time and to further control the balance of the human functions (homeostasis). 

Mimicking skin sensorial properties, the development of = “electronic skin” (e-skin) holds the promise of developing medical monitoring and highly sensitive prosthetic devices, biocompatible compliant medical implants, enhanced robotics, and more. The e-skin-related research field is a robust interdisciplinary approach, which combines micro-/nanoelectronics, material science, biotechnology, data transmission, and data processing technologies. The potential of epidermal electronics as biomimetic sensors [2], soft neural probes [3], prosthetics [4], implantable biomedical electronics [5], robotics [6], and a whole range of other skin-inspired devices [7] show great potential to change the world [8]. Its feasibility, however, relies on the desired e-skin characteristics such as flexibility, stretchability, self-healing ability, self-powering, biocompatibility, biodegradability and last, but not least, the reliability of large-scale manufacturing processes [9].

The present work revises e-skin development, provides an historical overview, describes the recent trends in material development and fabrication techniques, analyses some of the most promising health-oriented e-skin sensors, and finally addresses e-skin power-related management. Thoughts on future research trends and current limitations conclude the paper.

## 2. The Concept of E-Skin: A Short History and Schemata

### 2.1. E-Skin: From Fiction to Science

Science fiction works fancied the use of seamless prosthetics as early as E.A. Poe’s 1839 “*The Man That Was Used Up*”, where the Brevet Brigadier General John A. B. C. Smith was initially perceived as a “*truly fine-looking fellow*”, only to be later discovered as nothing but “*a large and exceedingly odd-looking bundle of something*”. The general’s body, severely mutilated in war and reduced to “the bundle of something” had to rely on prosthetic legs, arms, shoulders, bosom, teeth, eyes, and even an artificial palate and tongue. An interesting detail related to the seeing ability provided by the prosthetic eyes: “*you can’t imagine how well I see with the eyes of his make*”. When “reassembled”, it became apparent “*that the object […] was nothing more nor less than […] Brevet Brigadier General John A. B. C. Smith*”.

In the early 1950s, researchers explored the possibility of human/machine interfaces for prosthetics control, with first attempts to exploit the phantom-limb pain of amputees for motion control of motorized prostheses [10]. The 1960s introduced the “*artificial touch-sense*” when researchers used pressure transducers fitted on hand prostheses to generate stimuli. These developed sensors, applied to the skin with the help of electrodes [11] and implants, provided direct neural stimulation [12]. The feedback-based sensory systems, demonstrated in the 1970s, allowed proportional nerve stimulation and prosthesis control [13,14]. Essentially, the research on artificial touch with the development of robotic skins and mainstreaming of the touchscreen flourished in the 1980s [15]. In the 1990s, the advances in flexible materials, particularly polymers, such as polyimide (PI) [16] and polydimethylsiloxane (PDMS) [17], allowed the design of large surface-flexible circuits [18,19].

Furthermore, the early 2000s introduced the concept of electronic skin referred to as “sensitive skin” and was defined as “a large-area, flexible array of sensors with data processing capabilities, which can be used to cover the entire surface of a machine or even a part of a human body” [20]. Lumelsky et al. [21] presented an extensive overview of the first workshop dedicated to the electronic skin and was organized in 2000 by the NSA and DARPA. Consequently, the world of the e-skin expanded rapidly with some of the first stretchable metal electrodes presented in [22]. One step further, taken in 2014, demonstrated how the graphene-based transparent neural microelectrode arrays allowed simultaneous imaging and optogenetic neural stimulation [23]. A few years later, in 2018, Tybrandt et al. [3] used a composite material of gold-coated titanium dioxide nanowires in a silicone matrix and developed stretchable electrode grids applicable to long-term neural recording. A recent paper described a silk-based transparent e-skin for thermoregulation with a potential application in arthritis treatment [24]. Moreover, Gao et al. proposed a bifunctional temperature and pressure-imaging e-skin with self-healing capabilities [25]. The device integrated polyurethane and multi-walled carbon nanotubes on the same flexible cellulose nanocrystals carboxylated nitrile rubber polyethyleneimine (CNC XNBR) substrate.

### 2.2. The Concept of E-Skin Systems

**Figure 1:**
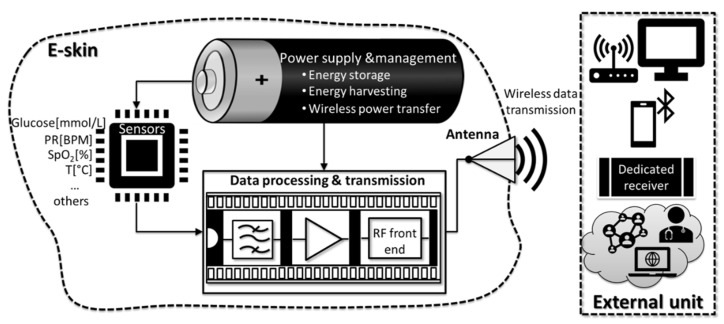
Working principle of an e-skin system.

The currently accepted concept of e-skin implemented into research can be described in Figure 1, which provides the overview of the main building blocks of an e-skin system. The first one, the sensing block, picked up the relevant biological stimuli, such as blood glucose levels, pulse rate (PR), peripheral capillary oxygen saturation (SpO_2_), temperature, and translated their magnitude into a measurable electrical signal. The next element, a data processing and transmission unit, including filters, amplifiers, and a radiofrequency (RF) front-end, could be inserted directly into the e-skin. This unit collected the analogue signals from the sensor block and performed the initial processing of the signal. The RF front-end generated and modulated an RF signal, with the output usually connected to an antenna for wireless data transmission. The data were then relayed to an external unit (dedicated receiver, mobile phone, cloud, or other means) using Wi-Fi, Bluetooth, or near field communication (NFC). All blocks were supplied with energy by the power supply and management block, which used an exchangeable/rechargeable battery, wireless power transfer (WPT) or performed energy harvesting in a self-powered scenario. 

## 3. Materials and Fabrication Methods

### 3.1. General Considerations

Developing mechanically flexible and stretchable materials similar to the human epidermis was challenging when the fabrication of high-performance e-skin sensors was attempted. These materials must allow and maintain intimate contact between devices and dynamically structured human skin or complex machine surfaces (for robotics applications) [26]. Moreover, developing patterning and assembling technologies of these materials to fabricate classical electronic components is crucial for building stretchable electronic systems. In this direction, the microfabrication technologies developed for rigid materials needed to be modified to obtain electronic devices capable of withstanding torsions, elongations, and compressions with preserved electrical functions. If the monitoring of biological parameters was the desired application of the designed e-skin sensors, wearability and biocompatibility were the essential features. Therefore, the sensors must not cause discomfort, irritation, or local sweating over that targeted attachment period to the skin. Wang et al. reported that thinner and softer sensors with smaller contact pressure between substrate and epidermis were more comfortable to wear [26].

Meanwhile, studies explored the potential use of biodegradable advanced 2D materials for health monitoring devices. Recently, ultra-thin, soft elastomeric materials demonstrated conformal contact with the skin surface. The performance of this class of integrated electronic e-skin sensors, mounted onto epidermis based only on van der Waals interactions, improved through increased contact area and fewer motion artefacts [27,28]. It has also been observed that the materials used to fabricate the e-skin sensors must present elastic moduli between 0.5 and 1.95 MPa and a stretchability of more than 140% [29].

In conclusion, the main “building blocks” of the e-skin electronic components such as substrate, conductors, semiconductors, and dielectrics must meet specific requirements: they should bend, twist or stretch without modifying functional properties and electronic performance during operation. Table 1 presented a summary of the materials used in e-skin sensors.

### 3.2. Substrates

Developing e-skin sensors required selection or synthesis of highly flexible substrates for materials’ design. Decreasing the elastic modulus of substrates could lead to an increased comfort level when wearing the e-skin sensors. Therefore, the materials used as substrates should have native or induced flexibility and stretchability upon using uni—or biaxial strain to generate microstructures in the deposited material. In this way, generating wrinkled and waved metal foils or silicon ribbons could produce stretchable substrates. Moreover, the first cycle of strain and release controlled the reversible stretchability range of the substrate. Intrinsically stretchable polymers as substrates for e-skin sensors were used due to their stretchability and stability. These properties ensured conformal integration of electronic components on the anatomic surface of the skin while avoiding mechanical degradation of the sensor during real-time operation. Moreover, the organic substrates, easy to integrate with conducting and semiconducting polymers, offered various chemical functionality for electrical conduction and provided suitable mechanical properties. Furthermore, breaking and rebuilding electrostatic interactions and hydrogen bonds within these materials dissipated induced strain [30]. The most used stretchable polymeric materials for e-skin substrate were: Poly(dimethylsiloxane) (PDMS) [31,32,33,34], other silicone rubber films [35,36], polyurethane (PU) [37,38,39], polyimide (PI) [40,41], polyethylene terephthalate (PET) [42,43], silk fabric-derived carbon textile [44], parylene [45,46,47], and even paper [48].

PDMS, a commercially available silicone rubber, is by far the most commonly used stretchable polymer substrate due to its manufacturability, affordable price, mechanical properties (Young modulus of 0.4–3.5 MPa), low dielectric constant-2.7 [49], biocompatibility, and chemical inertness. It is micropatternable [50] and can be obtained through the traditional or soft-lithography process, even from molded silk textiles [51] or leaves [50] or by spinning as thin substrates. However, the delamination from the skin due to sweat or moisture and the mismatch in Young’s moduli between PDMS and the human skin (25–220 kPa) were possible drawbacks of the method. Other silicon rubbers, such as Ecoflex, Dragon Skin, and Silbione, were considered biocompatible and allowed elongations up to 1000%. For example, Ecoflex with Young’s modulus of ~0.1 MPa and 1000% elongation at break, was suitable for large strain sensors [52].

On the other hand, PU showed excellent flexibility and elasticity and was used as a substrate or matrix for different composites. A thermoplastic PU fibrous mat, obtained through the electrospinning technique, constructed an aligned 3D conductive network with reduced graphene oxide (RGO) nanosheets [53]. Moreover, PU served as a stretchable substrate for Cu@Ag alloy nanowires forming an electrode with high electromechanical stability [54].

Sekitani’s team reported in [55] that PI presented an excellent stability under extreme bending. They fabricated organic thin-film transistors and complementary circuits on a thin PI foil (12.5 μm-thick) coated with a 500-nm-thick PI planarization layer, which could maintain circuits function, even when folded, at a radius of 100 μm. Takei’s group [56] also developed graphene kirigami structures on a PI film for a strain sensor that proved high stability and excellent reversibility. 

PET layers are frequently used as substrates for different conductive materials such as nanofibers [57], thin indium-tin-oxide (ITO) [58], graphite or with graphite/PEDOT:PSS [59], but it can suffer significant damage when highly bent [60].

Meanwhile, silk can be used as a substrate on e-skin sensors because it is biocompatible, flexible, and comfortable [44].

Parylene is a thermoplastic polymer deposited via chemical vapor deposition (CVD) in thin and pinhole-free films [46]. Parylene material is also known for its chemical inertness [45], biocompatibility, and low permeability to moisture. As a result, Parylene was already used for implantable [61] and MEMS devices [62] on a large scale. For example, Bae et al. developed a sensor with Parylene C substrate, which detected temperature and pressure simultaneously [47]. Kirigami’s technique was used to deposit gold electrodes on Parylene C in a pressure sensor [63].

Low-cost materials, or even recyclable materials, can be used as substrates. Recently, a wearable health monitoring device fabricated through an easy fabrication process integrated insulating recyclable and flexible papers as multiple functional layers [64]. This 3D stackable architecture device with a high deformability and conformability integrated flexible electronics within the insulating paper substrates for monitoring temperature, blood pressure, heart rate, and skin hydration. 

### 3.3. Conductive Materials and Interconnects

**Figure 2:**
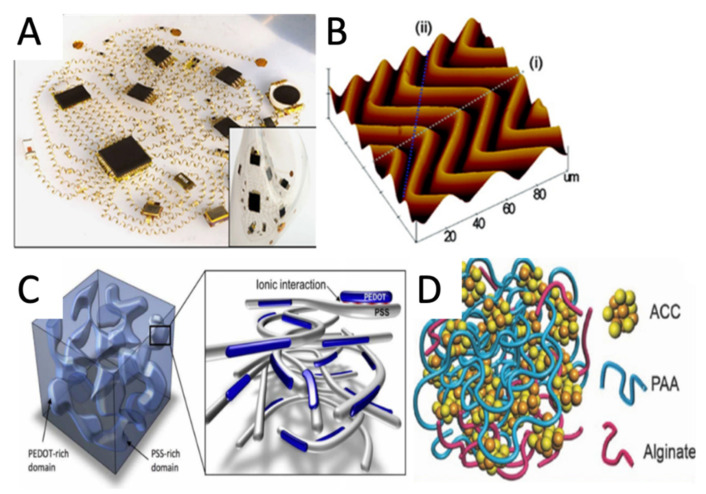
Overview of the conductive materials/methods used for e-skin: (**A**) Network of helical coils as electrical interconnects for soft electronics. Reprinted with permission from [94]; (**B**) AFM image of 2D “wavy” Si nanomembranes on PDMS substrate, reprinted with permission from [66]; (**C**) Schematic diagram of the morphology of a typical PEDOT:PSS film. Reprinted with permission from [67]; (**D**) Schematic structure of the ACC/PAA/alginate mineral hydrogel. Reprinted with permission from [70].

Different techniques have evolved to develop the conductive connections of e-skin sensors, such as:

- the “engineering” of rigid conductive materials (i.e., metallic serpentine as illustrated in (Figure 2A) to withstand elongation, compression, and twisting,
- the use of intrinsically stretchable conductors (conductive polymers, hydrogels, and ionogels),
- the formation of bulk composites of conductive materials in dielectric elastomers.

Conventional thick and brittle conductive electrodes (e.g., Au, Cu, Ag) without the mechanical characteristics cannot be elements of e-skin sensors. In 2003, Lacour et al. [22] developed the first stretchable electrodes depositing a 100-nm gold film on a 1-mm-thick PDMS membrane with built-in compressive stress to form surface waves. They reported that the metal film on an elastomer substrate could be stretched beyond the fracture strain of a freestanding metal film while remaining electrically conductive. Rogers’ group, in 2008, took a step forward and reported the fabrication of silicon ultra-thin flexible films (100 nm) for high-performance electronics [65]. Using a stretchable polymeric substrate coupled with a “rigid” conductor element, either on the surface or incorporated into the mass of the polymer as fillers, achieved the conductors’ flexibility and stretchability. Fabricating of stretchable electrodes and interconnections from the conductive polymer was possible when forming a partially delaminated wavy or non-coplanar geometry (Figure 2B) [66].

Conductive polymers helped reach a compromise between electrical properties and flexibility. They met the flexibility required e-skins, but had much lower electrical conductivity than metals such as Cu, Al, Ag, or Au. Developing new conductive polymeric materials enabled simpler fabrication processes (direct printing and coating) of mechanically robust devices with more intimate contact with skin. Conversely, highly conductive materials like poly(3,4-ethylenedioxythiophene) polystyrene sulfonate (PEDOT:PSS) could significantly improve their low stretchability upon the addition of non-ionic plasticisers [67] (Figure 2C) or fluorosurfactants [68]. Conductive polymers could also be buckled to obtain stretchable electrodes for knitted and wearable sensors [69]. However, with all the improvements, the organic-based stretchable conductors (such as PEDOT:PSS) remained inferior to metal conductors in terms of electrical conductivity. Achieving simultaneous high stretchability, conductivity, and reliability during operation in a conductive polymer is still a challenge. 

*Conductive hydrogels*, consisting of complex networks of two or three chemically or physically linked polymers, were promising materials for e-skin sensors due to their high stretchability. Moreover, most of them were also biocompatible, and the newly synthesized hydrogels showed improved mechanical properties [70]. For example, the synthetic elastic hydrogels composed of ~90% water and networks of polymers, forming ionic bonds and covalent crosslinks achieved stretches of 10–20 [71]. The covalent network preserved the memory of the initial state and allowed the hydrogel to return to its original shape after the stress stopped. However, in an elastic gel, the notches could partially reduce the stretchability: it could still be stretched beyond 20 times its initial length [72]. Other characteristics of hydrogels were considered when analyzing their potential as e-skin. For instance, bio-inspired hydrogel, based on calcium carbonate nanoparticles physically cross-linked by poly(acrylic acid) and alginate chains, was more attractive because of the self-repair and the ability to maintain ionic conductivity strains up to 10 times (Figure 2D) [70]. Adding humectants remedied the drawbacks, such as dehydration by evaporation [69]. Furthermore, the higher resistivity of ionic conductors, and the large stretchability, compensated for lower sheet resistance [73]. However, hydrogels or ionic gels based on chemically crosslinked polyacrylamide (PAAm) cannot maintain intimate and prolonged contact with the skin [74]. Recently, Wu et al. [75] introduced ethylene glycol(Eg)/glycerol (Gly) in a polyacrylamide/carrageenan DN hydrogel through a partial water replacement method, and achieved unprecedented thermal stability, avoiding dehydration at 70 °C and freezing at −18 °C. The newly acquired thermal stability, enhanced mechanical strength and stretchability, were attributed to strong hydrogen bonds between Eg/Gly and water molecules.

Conductive aerogels belong to a new class of materials with promising properties, including good electrical conductivity, low density, high specific surface area, and high porosity [76]. Several types of materials were proposed: MWCNTs/graphene aerogel [77], single-walled carbon nanotube aerogel [78], emulsion-templated silver nanowire aerogel [79], copper nanowire aerogel monoliths [76], and graphene foam [80].

Composite materials, the most researched active candidates for e-skin sensors, were obtained by combining conductive fillers with an insulating polymeric matrix. A filler concentration exceeding a certain threshold created a continuous conductive network of junctions between adjacent conductive fillers. Since fillers were aggregated into bundles and sank to the bottom during the polymer composites’ synthesis, using dispersants facilitated a better dispersion of the fillers [81]. Irrecoverable junctions between fillers increased the electrical resistance after stretching electrically conductive polymer composites. Carbon nanotubes (CNTs) in elastomers [82,83,84], graphene in PDMS [85], or Ag flake–polyurethane ink on polyurethane substrate [86], are conductive composites that proved to have a high stretchability and strain-tolerant conductivity. Metal nanowires (Ag–Au core-sheath nanowire in an elastomeric block copolymer matrix composite [87], metal nanoparticles (Ag NP in fluorine rubbers) [88]), and nanoflakes hybrid materials (Ag flake/Ag nanocrystals) in a PDMS elastomer presented similar properties [89]. Designing conductive materials for electrode deposition aimed at e-skin sensors also employed CNTs and graphene due to their high conductivity (long mean-free path of electrons) [90]. Moreover, the networks of nanotubes with isotropic orientations and graphene sheets allowed a certain degree of stretching without altering the film conductivity [31]. Kim et al. [91] reported a highly stretchable PU–Au nanoparticle conductor comprising Au NP (up to 485% strain), aligned under stretching to form the conductive pathways. A sensitive strain sensor comprised multiple ~20 nm thick layers of gold nanosheets deposited on an Ecoflex substrate [92]. Ultrathin gold nanowire-impregnated tissue paper was positioned between two thin PDMS sheets [93] in a sandwich-type architecture, resulting in a highly sensitive, flexible pressure sensor. Another proposed conductive composite was an ultralong Au-coated silver nanowire in an elastomeric block copolymer matrix. The thick Au shell prevented oxidation, and Ag ions leakage, improved the biocompatibility and maintained the conductive qualities of the composite [87]. In another study, Tian et al. [79] assembled 1D Ag nanowires (Ag NWs) in a hierarchical architecture and fabricated an Ag NW-based aerogel. This metal filer-aerogel composite exhibited high stretchability and high conductivity at a significantly low concentration of Ag NWs. 

Liquid metals represent another direction of developing e-skin conductive materials. For instance, Ga-based liquid metals were patterned and encapsulated into channels of elastomer substrates for e-skins. Liquid metals provide very high stretchability and can be used as electrodes and interconnect for compliant electrical circuits and e-skin connections [95,96]. Moreover, eutectic GaIn’ unique self-healing properties showed potential as a self-healing e-skin sensor [97].

### 3.4. Semiconductors

In an e-skin sensor, the rigid sensing materials used may diminish the stretchability and flexibility of the entire configuration. Therefore, selecting a sensing material should consider the mechanical, electrical, optical properties and the possibility to form different structures that facilitate the detection capacity. Recently, intense efforts focused on developing new intrinsically stretchable organic semiconductors for organic field-effect transistors (OFETs), organic light-emitting diodes (OLEDs), and organic solar cells (OSCs). Two major classes of substances were used as sensing materials in e-skin sensors: organic semiconductors and metal oxide semiconductors. The print-compatible and solution-processable solutions belonging to the π-conjugated organic semiconductors were selected. Pentacene, a relatively high mobile organic semiconductor, was one of the most used for sensors. It was thermally stable and deposited by thermal evaporation to produce channel layers for OFET-type devices [98,99,100]. Meanwhile, Noguchi et al. in [101] described a spatial distribution of the applied pressure read by reported 2025 pentacene FET active matrices from flexible pressure sensors of pressure-sensitive rubber. Other organic semiconductor materials for pressure sensing were polyvinylidene fluoride (PVDF) [102,103,104] and its copolymer trifluoroethylene, P(VDF-TrFE) [105] due to their fast-electro-mechanical responses and low-cost processes. However, the mobility of organic-based transistors must improve to achieve low-power operation.

Perovskite—piezoelectric materials, Pb(Zr, Ti)O_3_ (PZT), and BaTiO_3_ (BTO), having excellent piezoelectric properties, could be employed as self-powered sensors. Zhang et al. [106] proposed a flexible pressure sensor through microstructured PZT thin film deposition on a flexible PDMS substrate by a low-temperature pulsed laser deposition (PLD) method. This ultra-high detection-sensitive piezoresistive pressure sensor (~5.82 kPa^−1^) with a low degree of hysteresis (DH, ~0.45%) withstood a fatigue test when subjected to 10,000 cycles. Yang et al. [107] used another piezoelectric material—aTiO_3_—to obtain a composite consisting of polydopamine-modified BaTiO_3_ nanoparticles in a flexible PVDF matrix for a malleable piezoelectric pressure sensor. The tests performed on this sensor showed an enhanced piezoelectric output of the sensors due to the excellent dispersion of BTO particles in the PVDF polymer. However, the piezoelectric materials’ low flexibility and fatigue during long-term use limited their potential as wearable electronics.

Inorganic metal oxide semiconductors were employed to build UV photodetectors, tensile sensors, and nanogenerators. The photodetectors used various metal oxides, including zinc oxide (ZnO) [108,109], tin oxide (SnO_2_) [110], and titanium dioxide (TiO_2_) [111], as semiconductors. For instance, a Kevlar microfiber-ZnO nanowire hybrid structure incorporated into a wearable piezoelectric nanogenerator (PENG) [112] could produce energy for e-skin devices. ZnO NWs were hydrothermally grown from a seeding layer of ZnO on Kevlar microfiber. Ha et al. [113] reported hierarchical micro- and nano-structured ZnO nanowire arrays grown on a layer of microstructured PDMS in an interlocked geometry and with piezoresistive and piezoelectric sensing potential. The proposed structures permitted a stress-sensitive difference in the contact area between the interlocked ZnO NWs and an efficient bending of ZnO NWs to allow static and dynamic tactile stimuli detection. ZnO NWs deposited on a highly stretchable PU textile [109] were used for UV fast detection.

Furthermore, CNTs were used as active-matrix material [114] due to their excellent chemical and physical properties and tunable metallic/semiconducting properties. However, the integration of CNTs into flexible devices was complex because of poor control of chirality in CNTs. Despite their drawbacks, inorganic nanomaterials as active matrixes obtained by macroscale procedures (printing and transfer processes) offered high mobility and stability [115].

### 3.5. Encapsulation/Passivation

It is acknowledged that most organic semiconductor materials were vulnerable to water, oxygen, heat, chemicals, and light. Therefore, the encapsulating layer should have low water permeability or must be hydrophobic material. The encapsulation coating must also adhere well to the base material for good lasting protection of the entire sensor and error-free sensor operation. These characteristics defined passivation, and PDMS to be often used for passivation due to its flexibility and biocompatibility. However, it was permeable to water and some organic molecules [49]. Therefore, Ortigoza-Diaz et al. proposed Parylene with low permeability to moisture for multilayer coating and surface permeability tunning [46]. Compared with the traditional microstructured PDMS dielectric layer, the thermoplastic PU nanomembranes (TPUNM) deposited through the electrospinning process for the dielectric layer [116] was cheaper and was produced on a large scale. TPUNM with many gaps ranging from tens of nm to several µm allowed gas exchange. Furthermore, depositing a hybrid structure of a metal layer (Au, 200 nm thick) sandwiched between two polymer layers (parylene, 300 nm and 12.5 µm-thick) achieved the encapsulation and improved the air stability of the devices and circuits [55]. Nguyen et al. used Ecoflex 0030 as an encapsulation layer on a wrinkled metallic thin film-based soft strain sensor and succeeded to improve, in this way, the stretchability (260% elongation) and dynamic range (~50%) of the sensor [117]. Furthermore, the entire sensor assembly improved its stability during sharp bending, when the encapsulation layer had the same thickness as the elastomer substrate [55].

**Table 1:** Example of the materials used for e-skin sensors.

	Materials		Fabrication	Application	Properties/Obs.	Ref.
**Substrates**	Polymers	PDMS layer	PDMS molded from vinyl record	- self-powered tactile sensor	50 μm thick	[54]
		PI layer	Spin coating	photo-transistor	-	[118]
		Parylene C	chemical vapor deposition	- temperature/pressure sensor	3 µm thick	[47]
	Textile	Silk	- carbonization (Ar + H_2_)	- sweat sensor	- good uniformity and high mechanical strength	[44]
	Paper	Post-it Note paper	- used as received	- paper watch	- low-cost	[64]
**Conductors**	Conductor on elastomer	- system of Au waves on PDMS	- electron beam evaporation of Au onto warm PDMS	- conduction path	50-nm-thick layers of gold with a 5-nm adhesion interlayer of titanium or chromium	[21]
	Conductor on elastomer	- wrinkled gold stripes on PDMS	- electron beam evaporation through a shadow mask	- elastic interconnects	- buckled Au stripes (100-nm-thick) - retain electrical continuity when stretched up to 22%	[22]
	Conductive polymers	- interconnected hollow-sphere structures of polypyrrole (PPy) - sandwich electrods: copper foil/ITO-coated PET sheet	- emulsion method - casting process	- pressure sensors	active layer is both conductive and elastic 0.5 S cm^−1^	[43]
	Mussel-inspired hydrogel	- hydrogel formed by tannic acid-coated cellulose nanocrystals, poly(acrylic acid), and metal ions	- free radical polymerization - soaking in metal ions solution	- motions sensor	- stretching up to 2900% - toughness 5.60 MJ/m^3^ - self-healing properties and self-adhesive	[119]
	Organo-hydrogel	- polyacrylamide/carrageenan DN hydrogel modified with glycerol and ethylene glycol	- polymerization method - partial replacement of water with glycerol and ethylene glycol	- thermistor	- 1103% strain - high thermal stability - self-healing and high transparency	[75]
	Ionogel	(PAMPS)-based double networks gel filled with 66.4 wt% ionic liquid IL ([EMIm][DCA]) (520 μm)	- polymerization and IL wetting	- self-powered tactile sensor	- very sensitive - stretchability 121%	[54]
	Aerogel composite	- reduced graphene oxide (rGO) aerogel combined with PI	- freeze casting and thermal annealing	- strain sensor	- superflexible 3D architecture - low density	[80]
	Aerogel composite	- Ag nanowire-based aerogel with PDMS infiltration	- emulsion-template method - PDMS casting	-	- Ag: 50 mg cm^−3^ - high conductivity (65.7 S cm^−1^) - good stretchability (130% strain)	[79]
	Hybrid Composite	tough hydrogel laminar composite Ag flakes (1.9 μm length) in Ecoflex composite (40 μm) 80 wt% Active layer: PEDOT and Au pyramids on PDMS	- -transfer method with water-soluble 3M tape - screen printing - elastic conductor transfer method	- pressure sensor	- -conformal contact with the skin - elongation at break of 1780% - 501 Ω	[120]
	Laminar composite	*SWCNT laminated on PDMS* vertically aligned SWCNT (very sparse) films connected at one end;	- liquid-induced collapse of SWCNT films	- motion detector	- SWCNTs highly packed (42%) films fractured into gaps and islands, and bundles bridging the gaps - 280% strain, - 10,000 cycle durability	[121]
	Laminar composite	- two face-to-face AgNW (2%)-PI	- PI and Ag/PI spin-coating selective wet etching	- pressure sensor	- sensitivity 1.329 kPa^−1^ - partly embedded Ag NW	[122]
	Laminar composite	- sensing layer: MXene coated silk fibroin nanofiber (MXene-SF) membrane - electrode layer: patterned MXene on silk fibroin nanofiber (MXene ink-SF) membrane	- wet chemistry synthesis of SF - dip-coating MXene deposition on SF membrane	- pressure sensor	- stability over 10,000 cycles - breathable, degradable - Elongation at break 11.5 ± 6.4% - sensitivity 298.4 kPa^−1^	[123]
	Laminar composite	- dielectrics: PDMS layers - electrodes: AgNW on paper	- spin-coating - hydrothermal synthesis and airbrush spraying	- pressure sensor	- sandwich structure - sensitivity 1 kPa^−1^	[124]
	Liquid metals	- interconnects: Galinstan (68.5% Ga, 21.5% In, 10% Sn) in micro channels patterned in PDMS	- -PDMS casting, patterning and shading - microchannels filling	- temperature/force sensor	- Young’s Modulus 0.36–0.87 MPa - temperature coefficient of resistance (TCR) 0.5%/°C at 40 °C	[125]
**Semiconductors**	organic semicond.	- pentacene based FETs	- vacuum sublimation	- pressure sensor	- 2025 pentacene FETs	[101]
	organic semicond.	- friction layers: PVDF/0.005 wt% AgNWs nanofibers mats (NFM) and ethyl cellulose NFM	- mold casting - electrospinning	- triboelectric nanogenerator pressure sensor	- breathable - stability ~7200 cycles - AgNWs improve the polarization of PVDF nanofibers	[126]
	Perovskite s	- microstructured PZT thin film	- low-temperature pulsed laser deposition	- motion sensor	- negligible hysteresis rate	[106]
	MOX	- active material: ZnO nanorods on graphene layer	- G: CVD, Au transfer method - ZnO: wet chemical method	- phototransistor	- stability over 10 k bending cycles - responsivity: 2.5 × 10^6^ AW^−1^ - photoconductive gain: 8.3 × 10^6^	[127]
**Encapsulation**	Dielectric	PDMS	Spin-coating	- triboelectric nanogenerator/tactile sensing	50 μm thick top and bottom layers	[54]
	Dielectric	PU nanomembranes	Electrospinning deposition	- pressure sensor	- allows gas exchange	[116]
	Dielectric	Ecoflex	Spin coating	- strain sensor	- improved stretchability and dynamic range	[117]

Legend: (PAMPS) poly(2-acrylamido-2-methyl-1-propanesulfonic acid); 81-ethyl-3-methylimidazolium dicyanamide ([EMIm][DCA]); polyvinylpyrrolidone (PVP); PVDF polyvinylidene fluoride; rGO reduced graphene oxide.

### 3.6. Trends in E-Skin’s Materials

Using self-healing materials could increase the lifetime of electronic devices that come into intimate contact with the skin surface and move in tandem with the skin [128]. It is acknowledged that long-term wearing might cause fatigue or accidental micro-cracks that damage the composite surfaces and spread throughout the substrate, conductors, or dielectric. In extrinsic self-healing composites, these microcracks can be healed, their propagation can be prevented, and thus the major structural damage could be avoided. To date, different methods of obtaining self-healing electronic materials were tested. For instance, using microcapsules released the healing agent in the form of a liquid monomer. This monomer’s polymerization initiated by the contact between the microcracks and the microcapsules was a catalytic process [129]. A fundamental condition for efficient self-healing was the homogeneous distribution of the microcapsules within the entire mass of the composite. Another method was incorporating conductive fillers into a self-healing network, a common approach to obtain self-healable electronic conductors. In intrinsic self-healing materials, the repair of the cracks in composites occurred through reversible covalent, non-covalent, and hydrogen bonds. For example, Song et al. reported dynamic Ag–S bonds between Ag from an AgNW aerogel and S from a sulfur-containing molecule in a ternary network hydrogel to obtain 93% healing under near-infrared (NIR) laser irradiation [130]. Markvicka et al. used liquid metal droplets (GaIn eutectic) evenly distributed in a soft, silicone elastomer [94,95]. By controlled pressure, the microcapsules broke and gathered to form pathways with high electrical conductivity. Lately, some progress was reported towards solutions to increase the potential of self-healing materials, even though at the expense of a decreased material’s stretchability [131].

Since these sensors are in close contact with the skin and could be worn for a long time, they should be biocompatible and comfortable. Therefore, materials with interconnected pores have been proposed as substrates for permeable wearable skin-like electronics. For example, Yang et al. [119] developed a poly(vinylidene fluoride) nanofiber membrane with hydrophobicity and breathability. Moreover, these nanofiber membranes (NM) obtained by electrospinning showed high porosity, flexibility, and smoothness. Therefore, they could be incorporated in light, breathable, and printable electronics.

### 3.7. Deposition Methods

In almost all e-skin applications, the substrate must comply with two main requirements: flexibility and stretchability. Usually, the selected substrate for the application defined the right technology for sensors, transistors, resistors, or any other necessary electronic components. E-skin device fabrication techniques belong to two major groups: (1) classical techniques based on conventional microfabrication processes such as photolithography, vacuum based-deposition technology, etching, and (2) printing techniques.

Although expensive (considering the required area of the e-skin device), conventional vacuum deposition (sputtering and e-beam) was the most-used technological process in the fabrication of thin-films (TF) for e-skin applications such as sensors, thin-film transistors (TFT) and flexible printed circuit boards (PCB). Significant advantages compensated for the financial-related issue. For example, the low temperature controlled the material growth. Furthermore, the technique offered the possibility of reactive deposition for unique materials such as AIN or PZT (Pb(Zr, Ti)O_3_) for piezoelectric sensors on flexible materials [132]. Metal-organic chemical vapor deposition (MOCVD) and Metal-organic molecular beam deposition (MOMBD) showed great technological importance in fabricating an extensive range of electronics, including sensors or rigid substrates for e-skin devices. Despite the fact that the temperatures used for the e-skin-dedicated substrates were relatively high [133], the processes are still used. An alternative, however, was considered: the low-temperature pulsed laser deposition (LT PLD). This physical vapor deposition (PVD) technique comprised a high-power ultrashort pulsed laser beam focused inside a vacuum chamber and used it to strike a target of the material to be deposited. The material was heated, vaporized from the target, and then deposited on a substrate facing the target as a thin film. Recent developments in this area [106] have demonstrated that this method could succeed in fabricating PZT thin-film pressure sensors. Spray pyrolysis was often used to deposition of doped ZnO thin film layers for TFT on flexible substrates [134,135]. Furthermore, the ultrasonic spray was used to deposit graphene materials on textile [136] and air spray to deposit conductive films (Ag NWs) [137].

Interestingly, most of the e-skin sensors do not require the resolution and performances of conventional microsensors. Consequently, once disposable e-skin sensors became the fabrication target, printing was proposed for low-cost and mass production of such devices. The printing techniques were also suitable for exploring new possibilities of materials processing for sensors and systems development, even on non-planar surfaces. It was acknowledged that such outcomes were reached with difficulty via the conventional wafer-based fabrication techniques [138]. Therefore, screen printing [139], inkjet printing [140], gravure printing [141], and air-jet printing [142] developed as the most frequently used methods for printing on flexible substrates. A laser-printing technique of liquid metal inks for manufacturing 3D structure on flexible substrate is presented in [118]. The liquid metal alloy was printed on a hydrophobic, deformable 3D surface (Ecoflex). The technique revealed great performance and stability being a cost-effective solution for e-skin sensors. Bian et al. presented an overview of the laser printing techniques [143] and underlined the unique advantages of the laser printing technology: non-contact process, high efficiency, processing from micro to macro scale, able to process both organic and inorganic materials. Furthermore, Liu et al. proposed a solution for screen printing of graphene-based highly conductive layers [144], with a printing ink consisting of a combination of exfoliated graphene powder and carbon black (as conductive filler). The achieved resolution was around 90 µm, while the conductivity of the material was 2.15 × 10^4^ S/m for a 7 µm-thick layer. The proposed solution was suitable for mass production. Since specific applications used either e-skin fabricated directly onto a 3D surface or with a specific appropriate curvature to be further attached to corresponding 3D structures, 3D printing of sensors, antennas and conductive traces are promising devices. For instance, Huang et al. [145] mentioned a 3D printed tactile sensor of an elastomer of graphene and PDMS, Adams et al. [53] presented a 3D printed antenna of silver nanoparticle, and Valentine et al. [146] presented PCB conductive flexible traces of highly stretchable thermoplastic polyurethane (TPU) and silver flakes.

A recent trend in e-skin fabrication is based on mask-free and chemical-free methods, which employ a laser to prepare graphene and fabricate graphene-based electronic skins. Furthermore, Xiong et al. [147] presented the technique of chemically derived graphene oxide (GO) preparation using a laser, while Ye et al. [148] reported further significant advances in mask-free created micro-patterns. 

## 4. Power Management Approaches

Implementing e-skin technology imposed a detailed examination of the entire system, not the sensors alone, meaning the power supply, the various sensors and actuators, and pathways for signal extraction and processing. 

**Figure 3:**
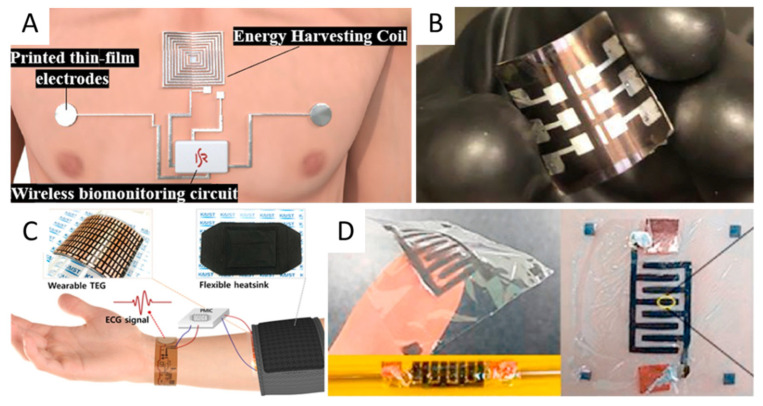
Examples of flexible energy harvesting and storage solutions: (**A**) wireless power transfer (WPT). Reprinted with permission from [149]; (**B**) flexible perovskite tandem photovoltaic cells. Reprinted with permission from [153]; (**C**) self-powered wearable electrocardiography using a wearable thermoelectric power generator. Reprinted with permission from [160]; (**D**) flexible planar micro-supercapacitor. Reprinted with permission from [172].

Wearable devices required enhanced portability and independence from interchangeable batteries. The main approaches considered for e-skin devices were based on self-powering schemes for long-term continuous use sensors or wireless power transfer (WPT) systems for on-demand data acquisition (Figure 3A). For instance, the electronic tattoos used a flexible Ag-In-Ga coil to receive up to 300 mW when placed directly on the skin and up to 100 mW if implanted [149]. 

Self-powering or autonomous e-skin devices employed energy harvesting schemes based on naturally available energy sources such as light, heat, movement, or biochemical elements to be harvested by dedicated transducers [150]. Furthermore, storing the excess energy in a battery or supercapacitor could achieve a continuous operation. Flexible photovoltaic cells demonstrated the highest reported power conversion efficiency (PCE) with a maximum of 30.8% for an InGaP-GaAs tandem solar cell [151]. Some of the best results obtained using other flexible thin-films showed a maximum PCE of 20.8% for Cu(In,Ga)Se_2_ (CIGS) solar cells grown on polymer substrates [152]. Other approaches included flexible solar cells based on perovskites with a record PCE of 21.3% obtained experimentally on a plastic substrate [153] (Figure 3B), organic materials with a PCE ~17% [154,155], quantum dots with maximum PCE reported to date of ~12% [156], and dye-sensitizer electrolytes with a maximum reported PCE of ~10% [157]. Although increasingly promising, limitations such as large area requirement and availability of direct light limited the applicability of the flexible photovoltaic cells as e-skin or made them usable only in combination with other energy sources.

The most promising flexible thermoelectric materials were based on polymers, carbon and hybrid materials [131]. The capacity of a thermoelectric (TE) to generate electrical energy was quantified through the “power factor” (PF = S^2^σ, where S is the Seebeck coefficient and σ is the electrical conductivity). One of the highest power factors reported for the organic TE material combination, PEDOT:PSS (Poly(3,4-ethylenedioxythiophene):Poly(styrenesulfonate)) was of 155 μWm^−1^K^−2^. It was achieved by including n-type MXene (Ti3C2Tx), an n-type 2D material [158]. A recent work reported on a combination of PEDOT:PSS polymer and a carbon nanotube (CNT) sheet, which reached a power factor of 30.54 μWm^−1^K^−2^ [159]. Typical problems for TE-powered e-skins were the relatively low power harvested directly from the human body and the difficulties in integrating it with other components; [160] was one of the few papers reporting a completely TE powered e-skin system (Figure 3C). The TE consisted of bismuth antimony telluride (Bi_2_Te_3_) grains assembled on a flexible polyimide film, which powered a temperature/humidity sensor, an accelerometer, a signal conditioning module, a microcontroller and an LCD module. This hybrid material indicated a power density of 3 μW cm^−2^ and a 10–15 mm bending radius.

The most common mechanical to electrical energy conversion processes were the piezoelectric effect and the triboelectric effect. In the first case, electrical charges were generated in a piezoelectric material when mechanical stress was applied. In the case of the triboelectric effect, electrical charges were generated because of the separation or friction of two different materials. The most common approaches used for piezoelectric generators for e-skin applications were based on ZnO nanostructures [161] (the Li-ZnO composite device successfully charged a capacitor with a power density of 0.45 W/cm^3^), lead zirconate titanate (PZT) films [162], and composite films containing barium titanate nanoparticles (BTO) [163] (reported power density of 0.76 μW cm^−2^). 

The first flexible triboelectric nanogenerator (TENG) was reported in 2012 by Wang et al. [164] The conversion of mechanical energy into electrical power was achieved through the friction of two polymers (Kapton and PET) with different triboelectric characteristics, stacked between two metal sheets (Au/Pd-Au). An output voltage of 3.3 V with a power density of ~10.4 mW cm^−3^ was reported. Since then, TENGs have been successfully implemented in e-skin applications, combining pressure- or strain-sensing abilities with self-powered capabilities. For example, a highly stretchable TENG-based e-skin with a sensitivity of 78.4 kPa^−1^, an open-circuit voltage of 202.4 V and an instantaneous power density of 6 mW m^−2^, at a strain of up to 200% was reported in [165]. The TENG is based on stacks of multilayered reduced graphene oxide (rGO)/silver nanowires (AgNWs) and thermoplastic polyurethane (TPU) mats [166]. The previously reported ultra-stretchable TENG presented an uniaxial strain of 1160%, an instantaneous peak power density of 35 mW/m^2^ and an output open-circuit voltage of 145 V, a low-pressure sensitivity of 0.013 kPa^−1^. The ultra-stretchability resulted from using ionic conductors (the Polyacrylamide (PAAm)-lithium chloride (LiCl) hydrogel) and PDMS. Polydimethylsiloxane (PDMS) films exhibiting high elasticity and biocompatibility were the most common choice for e-skin TENGs. A sensor-based on micro-frustum-array PDMS and (poly(vinylidenefluoride–trifluoroethylene) (P(VDF-TrFE)) nanofibers for pulse monitoring applications with a sensitivity of 5.67 V/10^5^ Pa was presented in [167]. The device was cycled 80,000 times and showed high accuracy of pulse waveforms in human test subjects. A self-powered, wireless-controlled e-skin composed of flexible photosensitive-triboelectric MAPbI3/PDMS units was used in [5] for neural modulation of a mouse brain hippocampus. A current of 8.94 nA, with a 0.659 V voltage, was recorded under dark conditions for a linear motor velocity of 0.04 m/s (used for controlled deformation of the e-skin), with a device-bending angle of 60°. 

Another emerging self-powering approach for e-skin systems was related to biochemical fuel cells (BFCs). In the case of bacterial BFCs, bacteria used as biocatalysts transformed the chemical energy of fluids, such as sweat or tears, into electrical power during the naturally occurring metabolic processes. Despite some concerns regarding possible microbial cytotoxicity, the bacterial BFCs promised desirable capabilities such as self-assembly, self-repair and self-maintenance. Recent work studied the use of naturally occurring Gram-positive skin bacteria *Staphylococcus epidermidis*, *Staphylococcus capitis*, *Micrococcus luteus* and Gram-negative ammonia-oxidizing bacteria species *Nitrosomonas europaea*, uncommon on human skin, for sweat-based power generation [168]. The three Gram-positive skin bacteria generated power densities of 36.23 ± 4.24 μW cm^−2^, 39.52 ± 5.05 μW cm^−2^, and 20.08 ± 2.28 μW cm^−2^, respectively. The Gram-negative ammonia-oxidizing bacteria species generated a comparable power density of 29.07 ± 2.00 μW cm^−2^. 

Enzymatic BFCs used enzymes as biocatalysts for power generation. The recently developed sweat-powered lactate BFCs were capable of multiplexed monitoring of key metabolic biomarkers (pH, glucose, ammonium and urea) and physical parameters (temperature, strain and pressure) [169]. The lactate BFC-powered e-skin system used the Bluetooth low energy (BLE) technology for wireless data transmission. The study reported the highest power density obtained in human sweat (3.5 mW/cm^2^) and demonstrated the long-term powering capability using repeated charging/discharging of a capacitor for about 60 hours. 

As far as flexible batteries are concerned, the main research directions focused on lithium (Li)-based batteries with flexible electrodes [170], supercapacitors [171,172] (Figure 3D) and combinations of traditional and carbon-based materials. One paper reported flexible electrodes for Li-ion batteries, which contained 92% silicon [173]. Carbon-coated silicon nanoparticles enclosed in conductive carbon nanotubes were kept in cellulose carbon nanosheets. The resulting flexible free-standing electrode had a silicon content of 85% and demonstrated a large capacity of 2710 mAh g^−1^ at a current of 0.2 A. Furthermore, the areal capacity reached a commercial-grade level of 5.58 mAh cm^−2^. Using textile cathodes allowed the combination of V_2_O_5_ hollow multi-shelled structures with conductive metallic fabric (Ni-cotton). The result was a capacitance of 222.4 mA h g^−1^ (mass loading of 2.5 mg cm^−2^) even after hundreds of bending/folding and charging/discharging cycles [174]. A flexible lithium transition metal oxide cathode was fabricated using LiMn_2_O_4_ nanocrystals on graphitic carbon nanofibers [175]. The resulting flexible 1D LiMn_2_O_4_-nanocarbon hybrid showed a capacity of 2.01 mAh cm^−2^ and a mass loading of 17.7 mg cm^−2^. Highly stretchable batteries were also reported, using zig-zag interconnects with gel cathodes and electrolytes [176]. The lithium-sulphur battery was stretched up to 420% and delivered an aerial capacity of 11.0 mAh cm^−2^ at a mass loading of 14 mg cm^−2^. One of the highest areal capacities reported to date for flexible lithium-ion batteries, 23 mAh cm^−2^, was achieved using aqueous Li-ion batteries based on LiTi_2_(PO_4_)_3_ (LTP) and LiMn_2_O_4_ (LMO) [177]. The interdigitated micro-supercapacitors with graphene-ink on thin polyimide and parylene films demonstrated high flexibility, a maximum areal capacitance of ~8.38 mF cm^−2^ at a scan rate of 100 mV/s, a specific capacitance of 22 F/g and a power density of 1.13 kW/kg. PDMS-based supercapacitors using nanowire hybrid conductors ((AgNW)/MnO_2_NW) recently showed a real specific capacitance of 371 mF cm^−2^ at a current density of 1 mA cm^−2^ while it extended to 160% [171]. As is the case of the complete e-skin system, developing flexible batteries focused on material research, areal capacitance, cycling reliability, flexibility and stretchability and self-healing abilities [178]. 

Data transfer from e-skin systems mainly relied on standard communication protocols such as RFID and near field communications (NFC) [179,180], Bluetooth Low Energy ([149,168]), and Wi-Fi ([181]), for short-range, medium-range and long-range transmission, respectively. However, on-demand wireless power transfer and data acquisition configuration, energy harvesting for continuous sensing and data transmission configuration, battery-powered configuration, or an energy harvesting/battery storage/on-demand data transfer combination were technology-dependent. 

## 5. Applications

Nowadays, the e-skin sensors designed and developed measure physiological variables like heart rate, blood oxygen saturation, glucose, or moisture and display them. Moreover, the capabilities of transparent [182] or semitransparent [183] layer-based devices extended for most of the sensing organs of the human body [184] to detect colorless and odorless gasses [185] vibration-, respiration-, sound- and pulse-changes [186]. The analysis of biomarkers and stimuli occurs in a network of e-skin sensors. For instance, a flexible sensor tag can long-term and noninvasively monitor the surface temperature for precise diagnostics and feedback treatment [187]. The dedicated sensing features rely on a large array of biomaterials, conventional or hybrid polymers grown on top of flexible and stretchable substrates, and must meet various requirements in terms of performance and multifunctionality [188]. Active matrix temperature sensor arrays [189] or passive sensors for temporary implants [190] are characterized by excellent sensitivity and stretchable reversibility. Thin-film materials deposition [191], and additive material deposition [192], could be done onto different substrates to enable sensing functions for precise quantification of the changing skin surface temperature. For such e-skin-mimic sensors, it is necessary to increase the sensitivity and resolution, reduce the detection limit, and expand the monitoring range (i.e., recognize temperature changes as small as 0.02 °C [193]). 

### 5.1. Strain/Pressure Sensors

**Figure 4:**
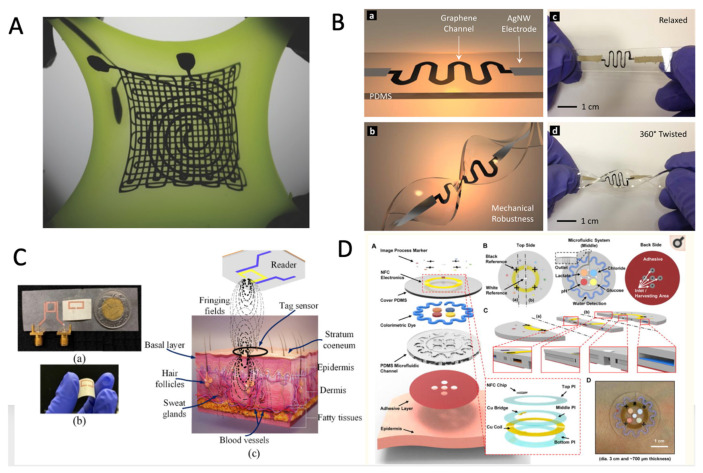
Examples of e-skin sensors: (**A**) Photograph of stretched strain/pressure sensor achieved using e3Dprinting. Reprinted with permission from [204] (**B**) stretchable graphene thermistors (a,b) Schematic diagram and (c,d) representative images of the stretchable graphene thermistors at relaxed and twisted states. Reprinted with permission from [283] (**C**) Operation of a glucose monitoring system: (a) Reader and the tag, (b) Tag flexibility, (c) Detailed illustration depicting the proposed application of this technology. The basal layer contains around 40% of ISF and according to its low distance from the sensor, it is the dominant layer determining the sensor response. Reprinted with permission from [257] (**D**) A wearable device for sweat sensing. Reprinted with permission from [269].

A unique and one of the most popular categories of e-skin sensors is the pressure/strain sensors (Figure 4A), dedicated to monitoring blood pressure and evaluating different activities. Flexible pressure/strain sensors convert the tactile stimulus into an electrical signal. From the construction point of view, several methods of converting the tactile stimuli could be described:

- piezoresistive,
- piezoelectric,
- capacitive,
- triboelectric,
- and optical.

Piezoresistive pressure sensors function on the variation of electrical resistance when a mechanical strain is applied. Choong et al. [194] proposed a stretchable resistive pressure sensor that employed micro-pyramid PDMS arrays with spring-like compressible platforms deposited to form a base for a conductive electrode. The sensor measured the primary features of a human pulse waveform. Their study introduced a stretchable electrode consisting of a conductive polymer—poly (3,4-ethyl-enedioxythiophene–poly (styrene-sulfonate) (PEDOT:PSS)—and an aqueous polyurethane dispersion (PUD) elastomer blend. The pyramidal structure enhanced the sensor’s pressure sensitivity resulting in a linear sensitivity of 4.88 kPa^−1^ over a wide range of pressures (from 0.37 to 5.9 kPa). Zhou et al. also reported similar microstructures in [195]. The group developed a flexible pressure sensor based on carbon powder/PDMS conductive layer and microdome structured PDMS membrane. The sensor presented a high sensitivity of 124 kPa^−1^ and a detection limit of 2 Pa. The sensor’s applications were related to health monitoring, soft robots, and human-machine interface technologies. Furthermore, Zhang et al. [196] developed a flexible pressure sensor using dome-like microstructures. They reported the sensors’ sensitivity of 6.61 kPa^−1^ in the pressure range 0–110 Pa, the detection limit of 1 Pa, the response time of 100 ms, and the stability over 3750 cycles of pressure loading/unloading. Furthermore, testing of e-skin sensors based on a microdome multilayer structure [197] showed a linear response between 0.0013–353 kPa, the pressure sensitivity of 47.7 kPa^−1^, a response time of 20 ms and high reliability: 5000 operating cycles of the pressure of 272 kPa. More information was provided by Park et al. when they studied piezoresistive pressure sensors with different microstructures [198]. The best pressure sensitivities were obtained for microdome structures: 47 kPa^−1^ (for the pressure range below 1 kPa), 90.6 kPa^−1^ (for 1 to 10 kPa), and 30.2 kPa^−1^ (for pressure range between 10 to 26 kPa). The potential application of the sensor included the detection of bio-signals such as human breath or artery/carotid pulse pressure. Zhong et al. [199] developed a P-HCF flexible pressure sensor with a micro-nano hybrid-structure and multistage sensing. From the fabrication perspective, various studies focused on mass printing. For instance, Maddipatla et al. used the inkjet printing technique [200] to fabricate a flexible strain gauge by printing Ag electrodes on paper substrate. The method was further optimized by Žlebič et al. [201], testing different Ag nanoparticle inks. Abu-Khalaf et al. [202] reported enhanced geometrical parameters of the inkjet-printed Ag nanoparticle traces on plasma-treated PDMS substrates. Zhang et al. [203] performed a comparative study of the parameters of two sensors: sensors made by the inkjet technique and sensors made by the screen-printing technique. The conclusion was that screen-printed sensors exhibited a gage factor (as the ratio of resistance change to applied strain) of 8.8 compared to 3.7 in the case of inkjet-printed sensors. The authors attributed the difference to the type of ink. Furthermore, Muth et al. [204] reported a method to fabricate piezoresistive strain sensors by embedded 3D printing (e-3DP). The results showed a gauge factor of 3.8 ± 0.6 and a stretchability of 100%. Kervran et al. [205] developed strain sensor-based arrays on a flexible substrate and measured the local deformations. They also reported a longitudinal gauge factor of −31 and a longitudinal piezoresistive coefficient of −4.1–10 Pa^−1^. The described how the process employed the n-type doped microcrystalline silicon (µc-Si) as piezoresistive material and deposited it directly on polyimide sheets at 165 °C.

It is acknowledged that the ability of a material to generate an electrical signal in response to an applied strain (by changing the distance between the dipoles or the length of the dipoles) allowed the development of piezoelectric pressure sensors. Moreover, to improve the imperfect sensor’ sensitivity, different geometries of the elastomer surface were proposed: pyramids, micropillars, waves, microdomes, and hierarchical structures. The conductive material can be filler type or deposited on the surface of the microstructured elastomer, thus increasing the contact area between the conductive components. Furthermore, carbon nanotube (CNT) materials under external pressure expressed piezoelectric effect, and Yee et al. [206] presented the fabrication techniques and performances of CNTs-based strain sensors in a critical review. Abot et al. [207] reported strain gauge sensor configurations using CNT wires, the numerical modelling of their piezoresistive response, and the parametric analysis schemes that determined the highest sensor sensitivity to mechanical loading. Another material with piezoelectric properties is graphene-woven fabrics (GFWs). Wang et al. [208] used GWFs for a flexible and wearable strain sensor assembled by adhering the GWFs on polymer and medical tape composite film. These graphene networks exhibited excellent performance under different tensile strains: relative resistance change could be 10 times at 2% strain, or 104 times at 8% strain and could be 0.07 times at 2% strain. The following signals were detected from the GWFs-PDMS-tape-based sensor: the current change was 5 nA at 30% strain, and the voltage change was ≈50 mV at 0.1% strain. The sensor’s membrane endured a significant deformation of 30% with completely reversible electrical properties. Dagdeviren et al. [209] proposed an array of pressure-sensing elements using rectangular patterns of the PZT ultrathin thin film on elastomer substrates. This highly sensitive device (~0.005 Pa) had serpentine traces for gate, drain and source and accommodated 30% uniaxial strain without significant influence on the sensing performance.

Capacitive pressure sensors’ function on the variation of the device’s capacity due to the change of the distance between two parallel-plate electrodes or the relative permittivity of the dielectric compared to the vacuum or of the electrodes’ surface area. Zhao et al. [210] presented a capacitance-based pressure sensor for imaging spatial pressure distributions. The sensor comprised Ag serpentine electrodes patterned on PET in a PDMS/Ag/Ecoflex/Ag/PDMS sandwich-structured array. It proved good performance having a detection limit of 6 Pa. Ruth et al. [211] reported that the micro-structuring of PDMS dielectrics into small pyramids improved their pressure sensitivity by over 30 times compared to unstructured PDMS layers of equivalent dimensions. The work presented a method to predict and design the pyramidal microstructure of the capacitive pressure sensors for the target pressure range.

Choi et al. proposed a linear and transparent capacitive pressure sensor for the pressure’s range of 5 to 100 kPa [212]. The sensor had a high resistance: under 5500 cycles at 100 kPa, the response time was 146 ms, and the transmittance was 85% at 550 nm. Cheng et al. [213] analyzed the capacitive pressure sensors’ behavior for different types and sizes of the dielectric microstructures. The study concluded that using large pyramid-type micro-structures with small distances between elements improved the sensor’s sensitivity and decreasing the size of micro-pyramids reduced hysteresis. The sensor retained a sensitivity of 3.73 kPa^−1^, a minimum detection limit of 0.1 Pa, and hysteresis of 4.42%. An optimization study [214]—through the COMSOL Multiphysics software—on the influence of different microstructures presented the results of simulations and showed that using the micropyramids produced the best performances. Schwartz et al. [215] combined a PDMS dielectric microstructure and the high-mobility semiconducting polyisoindigobithiophene-siloxane in a monolithic transistor design. This flexible pressure-sensitive organic thin film transistor had a maximum sensitivity of 8.4 kPa^−1^, a fast response time of <10 ms, high stability over >15,000 cycles and low power consumption of <1 mW. Kisic et al. [216] reported a capacitive pressure sensor, due to inkjet printing technology with a nanoparticle silver ink. The sensor had a sensitivity of 0.167 pF kPa^−1^ and measured pressure up to 100 kPa.

Optical pressure sensors converted a tactile signal into an electrical signal through light. Generally, the sensors included three components: a light source, a transmission medium, and a detector. Ramuz et al. [217] presented a flexible, stretchable, and transparent PDMS film, with a thickness of 600 µm, simultaneously functioning as waveguide and substrate. The fabrication process consisted of integrating an OLED (organic light-emitting diode) and an OPDs (organic photodiode into the PDMS substrate). Moreover, optical sensors, and especially flexible fiber optic sensors, provided an excellent solution to the practical challenges related to the e-skin sensors: the current leakage triggered by insufficient insulation, or the electromagnetic interference. [218] Guo et al. [219] pioneered a gel fiber that could carry up to 700% strain and be implanted. A proposed optical strain sensor based on dye-doped PDMS optical fiber [220] was characterized by the following parameters: linear and repeatable responses up to 100%, and the strain precision is below +/−1%. The same team presented a stretchable FBG-based optical (SFO) strain sensor, interrogated by a compact dual-comb, mode-locked fiber laser in free running [221]. This sensor model could be elongated to an axial strain up to 50% and showed sensitive response to tensile strain, bending, and torsion. Wang et al. [222] presented a graphene-added PDMS fiber with waterproof function and the following most important parameters: the tensile capacity of fiber 150%, the stretching cycle repeated 500 times, the optical loss coefficient of 2.58 dB/cm. The optic fiber sensors could be used to monitor muscle movements and breathing.

Triboelectric Nanogenerators (TENGs). Wang et al. [223] reviewed the progress of developing TENGs as self-powered active sensors and recognized four fundamental TENG modes: vertical contact-separation mode, lateral sliding mode, single electrode mode, and a freestanding triboelectric-layer mode. Furthermore, several studies discussed self-powered sensors and their specifications. For instance, Ren et al. [188] proposed a self-powered, high-resolution, and pressure-sensitive TESM (Triboelectric Sensor Matrice) based on single-electrode triboelectric generators. The results showed a linear response at pressures below 80 kPa, 10,000 cycles, response time: 70ms. Liu et al. [224] used the principle of traditional magnetic excitation generators and projected a self-charge excitation TENG towards high and stable output, while Yi et al. discussed a self-powered, single-electrode-based triboelectric sensor. The sensors detected the movement of a moving object/body in two dimensions and had a sensitivity of ≈887 pA/(cm s^−1^) [225].

To conclude, each conversion method of the tactile signal into an electrical signal had its pluses and minuses. The main advantages and disadvantages stemmed from the fabrication costs, technological complexity, sensitivity, or linearity. For example, the piezoresistive pressure sensor’s readout was more straightforward than that of the capacitive ones. Moreover, when using microstructures, the micropyramids and microdomes offer the best performance. Finally, the multilayer structures provide higher sensitivity and linearity of the sensor. Therefore, the selection of the measuring process depended on the intended application. A summary of the e-skin strain and pressure sensors is presented in Table 2.

**Table 2:** Summary of strain/pressure e-skin sensors.

Type	Materials	Sensibility	Elasticity	Max Press [kPa]	Resp. Time [ms]	Remarks	Ref.
**Piezo resistive**	carbon-based resistive ink/Ecoflex		100%			- G decreases by 50%. subjected to 1% strain for 1000 cycles, - G’, subjected to 100% strain for 1000 cycles, decreases by an order of magnitude;	[204]
	PEDOT:PSS/PUD/PDMS	4.88 kPa^−1^	50%/73 wt% PUD 100%/86 wt% PUD	0.37–5.9	200	- After 800 cycles of fatigue testing to 50% strain, the relative resistance of the 60% PUD was not able to recover to its initial value; - 3 types of stretchable electrodes tested (Au fabric electrode; a Ag fabric electrode; and electrospun poly(styrene-block-butadiene- block -styrene) nanofiber composite mat decorated with Ag NP) for stretchability and produced elongations of ∼10% (Au fabric), >40% (AgSBS mat), and >40% (Ag fabric, the highest stretchability).	[194]
	PDMS/silver nanoparticle ink		10% strain/straight line trace, AgNPs,1.8 mm line width 15%/horseshoe pattern/4 mm amplitude		-	- RSM-based DOE was performed, where the results indicated that the optimal parameters for the straight-line trace are one layer of silver NPs at a 1.78 mm line width. These parameters yielded up to 10% strain.	[202]
	rGO/PVDF	47.7 kPa^−1^		353	20	- 1.3 Pa minimum detection - fast response time	[226]
	PET/C-based polymer ink PET/silver NP-based ink,		1%		-	- The screen-printed strain sensors showed greater transverse sensitivities compared to the commercial foil gage - The printed sensors have significantly higher gage factors than standard foil gages and exhibited excellent linearity up to 0.4% strain with fatigue resistance up to 10 strain cycles.	[203]
	PET/silver NP-based ink		1500 micro-strains		-	- at 25 °C and 55% of relative humidity, after one month, sensor resistance values decrease with 1–1.5%, after two months, with 2–2.5% and after three months for 10%.	[201]
	*n*-type As doped μc-Si film/PI			7.10^3^	-	- Longitudinal gauge factors = −31 and longitudinal piezoresistive coefficients = −4 × 10^−10^ Pa^−1^; - Tests under tensile and compressive bending until radii of curvature down to 5 mm: no failure was observed.	[205]
	MWNT/PDMS	47 kPa^−1^(1 kPa) 90.6 kPa^−1^ (1–10 kPa) 30.2 kPa^−1^ (10–26 kPa)		30	12– 162	- The response time of interlocked composites with microdome, micropyramid, and micropillar arrays.	[198]
	P-HCF	26.6 kPa^−1^		0.02– 600	40	- The micro-pattern array is designed on the surfacemimicking the papillary lines on fingerprint surface to change pressure distribution to obtain linear sensitivity in whole sensing range.	[199]
	CPDMS	124 kPa^−1^ (0–200 Pa) 0.39 kPa^−1^ (0.2–12 kPa) 0.02 kPa^−1^ (12–50 kPa)		50		- detection limit of 2 Pa; - the relative resistance variation maintained around 10% over 1000 cycles with a 5% linear stretch.	[195]
	PDMS	6.61 kPa^−1^ (0–110 Pa)		16	100	- detection limit of 1 Pa;	[196]
**Triboelectric**	Au/PDMS/Ag	2.1 Pa			<5	- high sensitivity of 0.31 kPa^−1^, - fast response/relaxation time of <5 ms, - long-term stability/reliability of 30,000 cycles, - low detection limit of 2.1 Pa. - linear and high sensitivity region up to 40 kPa.	[227]
	Al/PDMS/Ag/PET	0.06 kPa^−1^		150	70	- linear response at pressures <80 kPa, - response time of 70 ms	[228]
	PET/PDMS/ITO	~13 mPa			120	- The power generation of the pyramid-featured device far surpassed that exhibited by the unstructured films and gave an output voltage of up to 18 V at a current density of ~0.13 μA/cm^2^. Furthermore, the as-prepared nanogenerator can be applied as a self-powered pressure sensor for sensing a water droplet and a falling feather (20 mg, ~0.4 Pa in contact pressure) with a low-end detection limit of ~13 mPa.	[229]
**Capacitive**	PI foils/Ag NP-based ink	0.167 pF kPa^−1^		100		- sensitivity of t 16.7 pF/bar for pressure range up to 1 bar and the obtained	[216]
	PDMS/ PEDOT:PSS	0.034 kPa^−1^		100	146	- transmittance over 85% - high linearity: R^2^ = 0.995	[212]
	PDMS	3.37 kPa^−1^		100	20	- ultralow detection limits: 0.1 Pa - hysteresis: ~4.42% - fast response	[213]
	PDMS/PiI2T-Si	8.4 kPa^−1^		60	10	- maximum sensitivity of 8.4 kPa^−1^, - fast response time, - high stability over >15,000 cycles - a low power consumption of <1 mW;	[215]

### 5.2. Temperature Sensors

The temperature sensors (Figure 4B) designed for local temperature monitoring of the human body must meet the general properties of the e-skin sensors: high level of flexibility and sensitivity, conformability, biocompatibility, and lightweight. Relevant classes of temperature sensors based on different detection mechanisms included:

- resistive temperature detectors (RTDs),
- positive- and negative-temperature coefficient thermistors (PTC and NTC thermistors),
- thermocouple temperature sensors, and
- integrated circuits (IC) temperature sensors [230].

Table 3 presents the appropriate classes of wearable temperature e-skin sensors with their characteristics.

Resistive temperature sensors (RTD). Since thermoresistive effects occurred in thin films metals, their oxides, and semiconductors as conventional active materials, the related temperature detectors were the most frequently used due to fair linearity, accuracy, repeatability, and high long-term stability. However, as they entered the self-heating regime, the detectors were generally used in domains that do not match the requirements for e-skin sensors. Moreover, they presented a temperature detection interval that was considerably larger than that of interest and converted thermal signals into electrical ones based on a quasi-linear temperature dependence on resistivity. Webb et al. [231] proposed an ultrathin conformal thermal sensor that relies on TCR value in thin (50 nm), narrow (20 μm) serpentine traces of gold, manufactured on nanoscale membranes of silicon (320 nm thick) employing lithographic techniques. Nonetheless, to avoid self-heating, the probe current was limited to 160 μA, which resulted in heating less than 0.02 °C, enough for inaccurate measurements. 

Positive- and negative-temperature coefficient thermistors. Monitoring the human temperature for healthcare purposes required high sensitivity sensors within the range of homeostasis. As passive components with a strong resistance dependence upon temperature, thermistors were classified based on positive (PTC) and negative (NTC) temperature coefficients. While silicon-based PTC thermistors are linear, NTC thermistors offered nonlinearity and, besides, are calibration-dependent. Like RTDs, thermistors converted the temperature changes into resistance changes with higher sensitivity and shorter response times. However, the persistent risk of self-heating and the rigidity of the frequently used materials constrained their performance as e-skin-mimic sensors. Jeon et al. [232] approached the latest challenge and used phase transformation from crystalline to amorphous to achieve a thermistor with much higher sensitivity on nickel microparticle-filled polymer composites. 

Nonetheless, a reproducible temperature-sensing response was difficult to reach. Yokota et al. also addressed the sensitivity aspect and reported printable PTC sensors of flexible ultrasensitive composite materials [233]. Their unique temperature sensors detected changes in resistivity by six orders of magnitude when the temperature changed by less than 5 °C. The sensor’s most sensitive range was within 25 °C to 50 °C, overlapping all meaningful physiological ranges in terms of temperature. Moreover, a similar flexible resistive temperature sensor that exhibited remarkable adhesion properties and was highly sensitive has been developed and reported [234]. 

Thermoelectric temperature sensors. Self-powered temperature sensors based on the thermoelectric effect converted temperature gradients into voltage signals. This effect manifested when carriers (electrons and holes) moved with a temperature gradient and caused a current flow. The recently developed organic thermoelectric materials presented the effect, and their applicability was further studied. For instance, Zhang et al. [235] developed a temperature sensor, flexible and accurate in resolution, suited for e-skin-mimic electronics. Furthermore, Jung et al. [236] used flexible and inexpensive organic and thermoelectric materials, nanoparticles, silicon rubber, and paper platforms and developed a bimodal sensor with temperature-sensing functions over a wide range (150 °C). The temperature sensor was the most appropriate for e-skin applications and could be tuned to a narrower temperature range. Moreover, thermoelectric sensors could supply power for other sensors in e-skin-mimic electronics.

Integrated circuits (IC) temperature sensors. IC temperature sensors anticipated temperature based on their dependence on silicon bandgap. A precision current spiked the internal forward-biased PN junction and resulted in a base-to-emitter voltage change (ΔV_BE_), equivalent to a temperature. Since the correspondence was predictable, the IC temperature sensors were highly linear and accurate (up to ±0.1 degrees C between −5 °C to 50 °C) across narrower-than RTDs or Thermistor temperature ranges. For instance, a flexible wireless e-skin system prototype, which incorporated a reconfigurable readout IC manufactured in a 0.18 μm CMOS process, was reported to have been developed [230]. The reported structure delivered three operating modes for each sensing function of the device. Emphasis was placed on the T mode, which detected temperature changes induced by resistance in the pyroresistive operation. For this purpose, conventional voltage conversion methods were replaced by frequency displacements as stated by resistance changes. 

Table 3 summarizes the e-skin temperature sensors from the literature.

**Table 3:** E-skin Temperature Sensors Characteristics.

E-Skin Temperature Sensitive Types	Working Mechanism	Linearity	Sensitivity (°C^−1^)		Response Time [ms]	Self-Heating	References
			Min	Max			
Thermo-resistivity	RTD	Fair	0.028%	2.09%	3.7–13.1	Yes	[231,237,238,239]
	Thermistors	Pour	0.80%	1.07%	1800–7000	Yes	[191,234,240]
Thermo-electricity	Thermoelectric	Fair	20 μV	45.3 μV	300	No	[38,236]
Solid state	Semiconductor IC	Best	10 mV 1 mA	20 mV 1 mA	-	Yes	[230]

### 5.3. Glucose Sensors

Specific devices for self-monitoring of glucose levels are essential for diabetes mellitus patients’ survival. Furthermore, these devices should provide noninvasive or minimal invasive measurements and accurate readings of any glucose variations in biofluids (i.e., blood, tears, saliva and interstitial fluid) while being user-friendly and accessible [241]. Since the accuracy and reproducibility of measurements are essential aspects, international standards certified and monitored the reference values and error grids (0–500 mg dL^−1^-corresponds to the blood glucose levels of patients with diabetes mellitus) [240,242]. 

The glucose sensors domain constantly evolved with new steps to design and fabricate flexible substrates (e-skin). A synthesis of the e-skin glucose sensors is presented in Table 4. However, the past years have demonstrated challenges that have affected the readings. For instance, the conventional method of pricking fingers, the most accurate widespread method of self-detection of glucose to date, is painful, uncomfortable and presents a significant risk of infection. Therefore, other solutions were targeted. From the sampling perspective, the family of glucose sensors included point sample and continuous monitoring with variations based on the contact of glucose sensors with the skin:

- invasive,
- minimally invasive; and
- noninvasive.

From the working-mechanism perspective, the commercial blood glucose devices were largely enzyme-based electrochemical sensors and involved enzyme-catalyzed reactions. For instance, the glucose oxidase on the testing strip facilitated gluconic acids from the glucose in the blood sample. The reaction with ferricyanide on the testing strip produced ferrocyanide, the base of the blood glucose reading [243]. A fluorescence-based hydrogel glucose invasive sensor is another example tested in vitro and in vivo (on pigs over 45 days) [244]. The device comprised two units: one implantable and one external. The detection unit consisted of the glucose-responsive fluorescence dye (GF-polyethylene glycol PEG-gel), a light-emitting diode (LED), and a photodiode (PD) mounted on a flexible substrate. The GF dye excited by the LED light and the intensity of the fluorescent response detected by the PD corresponded to the glucose concentration in the sample. The external component wirelessly transferred the signals for further recording. However, challenges such as controlling the gel swelling and immune responses during long term readings limited the subcutaneous insertion of the device. Consequently, minimally invasive methods emerged. 

Minimally invasive sensors attempted to minimize the patients’ injury, pain, and inconvenience during the collection of the necessary biofluids. Since the glucose concentration in the interstitial fluid follows the amplitude and dynamics of blood and plasma glucose [245], microneedles (MN) -single or array- of around 700 µm-height, microfabricated from different materials, were the main solution for minimally invasive options. For instance, Ribet et al. [246] proposed a planar amperometric glucose sensor, having a sensing area of approx. 0.04 mm^2^ and the insertion length lower than 1 mm (dermal region). The system’s advantage consisted of reduced sensor size and an optimal insertion into the superficial layers of skin for interstitial fluid extraction [247]. The proposed enzymatic biosensor presented three electrodes: a working electrode (WE), a platinum counter electrode (CE), and an IrOx quasi-reference electrode (Q-RE). The WE functionalized with glucose oxidase (GOx) catalyzed glucose molecules (target molecule) decomposition into H_2_O_2_. The electrochemical oxidization of H_2_O_2_ on the surface of the WE occurred at +0.6 V applied, and an electrical current proportional to the initial glucose concentration was recorded. The Q-RE kept the WE potential fixed and provided good biocompatibility, mechanical stability, and little long-term potential drift. Three other semipermeable membranes were deposited on the WE surface to avoid the oxidation of other interstitial fluids solutes such as uric acid (UA) or ascorbic acid (AA). The first membrane embedded the enzyme GOx, the second one was a permselective membrane with different diffusivity of glucose and oxygen through the polyurethane Selectophore™ (PU) layer, and the third membrane of 5 wt% Nafion excluded the anionic electroactive substrates, UA, and AA. The excellent biocompatibility made it appropriate for long term implantation [248]. The sensor’s sensitivity was 1.51 nA/mM in the linear range and was normalized to the WE area, to 12.7 μA·mM^−1^·cm^−2^ [246,249]. Since detection sensitivity and linearity must improve, Lee et al. [250] updated a minimally invasive glucose sensor with an arrangement of nanostructured layers that coated the electrodes. The amperometric sensor comprised an AuZnO_x_ layer that enhanced the catalytic oxidation of H_2_O_2_ and prevented the passivation of Au by chloride ions present in the biological samples. The MN array sensor was initially developed for glucose detection in cells. The presence of anionic species strongly adsorbed on the Au surface inhibited the electrochemical oxidation of glucose [251]. The AuZnO_x_ showed a four times higher sensitivity than an AuZn layer, indicating that the AuZnO_x_ layer improved glucose detection sensitivity and blocked chloride ions’ adsorption at approximately 80%. The proposed sensor was examined for glucose detection and toxicity. The glucose response showed two linear ranges, one from 0.01–5.0 mM because the adsorption process was dominant and the other from 5–50 mM since the diffusion control process dominated at the high concentration range [252]. The detection limit was determined to be 1.2 (±0.026) μM (95% confidence level). The toxicity test and single-cell analysis of the AuZnO_x_/pTCA-GO_x_/NF sensor assessed the biocompatibility of the sensing material for the in vivo applications. The results also showed that the proposed sensor could obtain detailed information from the cell analysis besides continuous glucose monitoring.

The fourth-generation glucose sensors involving direct electro-oxidation of glucose to gluconic acid via non-enzymatic electron transfer employed nanoporous material for electrodes array: a highly porous platinum black (Pt-black). The system comprised a fabricated Au/Pt black-nafion as the working electrode and the Au/Ag/AgCl-nafion counter/reference electrode in a three-electrode setup used for the amperometric measurements [253]. A 150 μm stainless-steel substrate supported the needles with a length and width of 650μm and 110 μm respectively [248]. The complex and well-defined dendritic structure was transformed into a porous construction after packaging with the Nafion ionomer (a biocompatible ionomer). Furthermore, Zhang et al. [254] proposed a patch system, which comprised dissolving and insulin-releasing MN to respond to blood glucose levels. The system was designed as minimally invasive insulin therapy for type 1 diabetes mellitus patients. The dissolving and biodegradable MN were fabricated from gelatin and starch and encapsulated insulin-releasing gold nanocluster (AuNC) nanocarriers. The patch consisted of MN arrays (11 × 11 conical MN per array) of needles with the following geometry: height of 756 μm, bottom diameter of 356 μm, tip diameter of 10 μm, and tip-to-tip distance of 591 μm. The AuNC nanocarriers were additives and improved the mechanical strength of the needles to enable the insertion of the MN into the skin, as demonstrated by the in vivo testing on mice. The insulin-loaded MN patches exhibited a higher rate of insulin release in diabetic subjects (9–20 mM), with a maximum drug release after 60–120 min compared with a lower concentration and relatively slower and less insulin release corresponding to healthy subjects (3–8 mM). Moreover, the insulin kinetics confirmed the preferential bonding of glucose to the phenylboronic acid molecules on the AuNC nanocomplex. In an in vivo one-time administration test, the patches exhibited reversible local skin irritation upon insertion and no systemic toxicity. Since clinical practice required glucose monitoring, developing sensors for continuous measurement was essential. For instance, Zhao et al. [255] reported an MN-based electrochemical biosensor. The device comprised silk/D-sorbitol pyramidal MN integrated with platinum (Pt) (RE) and silver (Ag) wires (CE), and glucose-selective enzymes (glucose oxidase, GOD WE) attached to the MN during fabrication. The silk/D-sorbitol composite provided a biocompatible environment for the enzyme molecules. The silk protected the device from temperature and moisture. The in vitro tests showed a quick response at low glucose concentrations with good linearity.

Another technique of detection that can be implemented for e-skin glucose sensors is based on measuring changes in reflected electromagnetic radiation transmitted through the skin. It is a direct relation of glucose concentration with the dielectric characteristics of tissue dependent on the resonant frequency. For instance, Kiani et al. [256] proposed a system for continuous glucose monitoring. It functioned on a microwave resonant sensor at two frequencies/channels of 5.5 and 8.5 GHz and with a quality factor of 180 and 106, respectively. The in vivo test (compared with a glucometer) showed a maximum error of about 3%. The sensor’s substrate integrated waveguide (SIW) had a relative permittivity of 3.55 and dimensions of 30 mm × 18 mm × 0.508 mm. The design created a difference in potential that increased a capacitive and an inductive contribution, the field distribution and resonance. At the range of glucose level changes (89–262 mg/dL), the frequency detection resolution (FDR) values for both channels used were 3.58 and 3.53 MHz/(mg/dL), respectively. The sensitivity of the proposed sensor was 0.04 and 0.061/(mg/dL), respectively. The FDR and the sensor’s free load resonance frequency, 8500 and 5500 MHz, respectively, defined the sensitivity for the channels used. FDR was defined as the rapport of ΔC (the glucose level variations) and ΔF (relative resonant frequency shift). 

Further on, Baghelani et al. [257] reported a noninvasive system for real-time monitoring of interstitial fluid glucose (Figure 4C). The system included a chip-less tag sensor applicable to the patients’ skin and a reader encased in a smartwatch. The electromagnetic couple between the tag and the reader powered the label sensor, making the system external and battery free. The detection accuracy was ~1 mM/l within the physiological range of glucose (the resonance frequency shift was 38 kHz).

**Table 4:** Summary of the main e-skin glucose sensors.

Method	Testing Model	Values				Remarks	Ref
		LOD (μM)	Sens. (μA mM^−1^ cm^−2^)	Linear Range	Resp. Time		
Optical	In vitro In vivo (rats and pigs)	NA.	fluorescence int. 103.0 ± 6.6% at 100 mg dL^−1^, 98.6 ± 3.5% at 300 mg dL^−1^, 101.2 ± 4.2% at 500 mg dL^−1^, 94.4 ± 5.4% at 1000 mg dL^−1^	0–500 mg/dL	90% of th max. fluorescence in 18 min	- Flexible and wearable structure - Minimally invasive procedure - Tested for 45 days	[244]
	In vitro In vivo (mice)		colour change (violet–blue–green with increasing glucose concentration)		5 min (reaction time)	- Flexible and wearable structure - Minimally invasive, painless, - Naked-eye glucose monitoring - Continuous glucose monitoring possible.	[258]
Electrochemical	in vitro		1.51 nA/mM 12.7	0–300 mg/dL		- Flexible structure - Minimally invasive procedure - The reaction time of 10 mg·dL^−^^1^·min^−^^1^~300 s	[246]
	In vivo	NA	2.44 nA/mM,	0–200 mg/dL		- Minimally invasive procedure in a laboratory under - A maximum sensing variation rate 910 mg·dL^−^^1^·min^−^^1^~300 s	[259]
	In vitro	1.2 (±0.026) μM.	NA	0.01–5 mM 5–50 mM	1 min	- Minimally invasive procedure - Maintain ~95% initial sensitivity over 30 days	[250]
	in-vitro	10 μM	5.786 μA mM^−1^ cm^−2^, detection limits,	(1–20 mM)	2 s	- Minimally invasive procedure - Moderate lifetime of 7 days - The toxicity of MN not studied - Stability (2% lost in 7 h), - Selectivity, reproducibility	[253]
	In-vitro in-vivo (rats)	22.5 μM	4.380 ± 0.2 μA mM^−1^ cm^−2^	1–20 mM			
				1.7–10.4 mM		- Quick response at low glucose concentrations	[255]
glucose-responsive insulin- releasing MN	In-vitro in-vivo				10 min	- AuNC drugs exhibited glucose-responsive insulin-releasing manners responding to different glucose levels, due to the preferred bonding of glucose to the phenylboronic acid molecules on the AuNC nanocomple	[254]
Microwave Resonant	In vitro In vivo (humans)		- Resonance frequencies in 5.5 & 8.5 GHz with quality factor of 180 and 106, - FDR = 3.58 MHz/(mg dL^−1^) and 3.53 MHz/(mg dL^−1^), - corresponding to 0.04 and 0.061/mg dL^−1^).	150–500 mg/dL	-	- Flexible and wearable structure - Noninvasive - No-extraction is required - Requires optimizing the readability of the reading mode and interpreting the measured signals - Communication with portable readout circuitry - should be investigated	[256]
	In vitro	5 mM/L	4 GHz 38 kHz/1 mM/L	0–40		- Flexible and wearable structure - Noninvasive - No-extraction is required - Experiments on mouse skin	[257]

### 5.4. Sweat-Based Biomarker Monitoring Wearable Systems

Sweat analysis on wearable platforms (Figure 4D) is currently considered as a viable alternative to invasive blood analysis due to the convenient collection of samples and continuous real-time monitoring of several body biochemical parameters. Sweat analysis via electrochemical (amperometric), colorimetric [260,261] or bioaffinity sensors [141,262] allowed the evaluation of specific physiological parameters for health monitoring and could be performed on variable e-skin platforms that may identify single or multiple analytes. For instance, an ion-selective potentiometric cell encased in a wearable tattoo platform permitted the monitoring of ammonium levels in sweat [263]. The skin-worn sensor, intended for sports performance and metabolic disorder evaluation, fabricated on a screen-printed layout, incorporated all-solid-state potentiometric sensor technology for the electrodes and a nonactin ionophore-based ammonium-selective polymeric membrane. However, the focus on e-skin as a multiplex system to detect concomitantly simple or complex molecules is essential and has become of great interest. Moreover, the partitioning of analytes [264] has been developed to become integrated into more complex devices as e-skin systems for easy to wear and noninvasive analysis of biomarkers. One example of a device for multiplexed in situ sweat analysis is the fully integrated wearable sensor for noninvasive monitoring of metabolites, electrolytes, and temperature [265]. The system, designed as a wristband and headband wearable during prolonged physical activities, had the advantage of conformal contact with the skin and in situ signal conditioning, processing, and wireless transmission. The platform comprised amperometric enzymatic sensors to detect glucose and lactate, ion-selective electrodes (ISEs) to detect Na^+^ and K^+^, and a resistance-based sensor to measure the temperature. Attention was paid to the system’s power autonomy and voltage, and reading stability. The device included (1) a Prussian blue dye mediator to cancel the reduction potentials and activate the sensors without an external power source, (2) poly(3,4-ethylenedioxythiophene) polystyrene sulfonate (PEDOT:PSS) as an ion-to-electron transducer in the ISEs and carbon nanotubes in the PVB reference membrane, and (3) Parylene as an electrically insulating layer between the metal lines and the sweaty skin. The platform included a chemical sensor array for real-time monitoring of NA^+^ during cycling exercises and concluded the potential of sweat as a key biomarker for acute dehydration. The results indicated that this system could improve the transition from the classic method [266] to the e-skin-based noninvasive sweat analysis. Further development of wearable sensors integrated onto a platform employed iontophoresis for autonomous sweat extraction to improve the sample size and accuracy [267]. In this case, Pilocarpine was administered to the targeted sweat glands via iontophoresis and contributed to on-demand sweat collection followed by in situ real-time detection of Na^+^, Cl^−^ and glucose. This system showed potential diagnostic value in cystic fibrosis and diabetes mellitus. Since there is also a need to collect and guide the sweat towards the sensing platform, Choi et al. introduced a thin, soft skin-like microfluidic device mounted to the skin capable of collecting, storing, and sequentially directing the sweat for chemical analysis. The studies on human subjects showed an accurate chemical analysis of lactate, sodium, and potassium concentrations and their temporal variations and demonstrated the clinical potential [268]. Another method to construct a soft wearable system was based on colorimetric detection and relied on the exposure of the target sweat analytes to reagents and consecutive measurable color changes upon their interactions. Koh et al. [269] used this principle and proposed one soft wearable microfluidic device capable of capturing, storing, and analyzing sweat. The ability of sweat to secrete other than naturally generated analytes explained the rapidly growing interest in developing designated sensors for analytes that are not usually expected, such as alcohol, or that could be related to pathology, such as heavy metals or drugs. It is possible to detect ethanol in as non-invasive manner as possible with a wearable tattoo-based biosensing device, which used iontophoresis to induce sweat and amperometry to detect the target analyte. The worn skin device comprised a flexible wireless system that controlled the processes and communicated wirelessly in real-time for continuous monitoring [270]. Moreover, identification of drugs [271,272] and heavy metals are for medical use as the body attempts to eject these as toxins in sweat [273]. Furthermore, a sensing platform may be used to diagnose various diseases. For instance, non-invasive continuous analysis of body fluids could monitor ionized calcium and pH levels and identify related diseases such as primary hyperparathyroidism and renal lithiasis. One wearable electrochemical device used a disposable and flexible array of sensors interfaced with a flexible printed circuit board for real-time quantification of Ca^2+^ and pH. The high repeatability and selectivity of the fabricated sensors demonstrated the platform’s potential as a non-invasive monitoring device [274]. Furthermore, developing a wearable multi-sensing flexible microfluidic platform allowed on-body testing of lactate, Na^+^, pH and temperature for internal calibration of human sweat. The platform, tested during cycle ergometry and treadmill running [275], wirelessly transmitted real-time data generated by the continuous flow of sweat that stimulated the sensors within: (1) the potentiometric Na^+^ sensors of polyvinyl chloride (PVC) functional membrane on Poly(3,4-ethylenedioxythiophene) (PEDOT) polymer, (2) the pH sensing layer on an iridium oxide (IrOx) membrane with high sensitivity, and (3) the amperometric-based lactate sensor of doped enzymes on a semipermeable copolymer membrane and surface polyurethane layers. Developing wearable technology could be applied to sports to identify the related chemical changes within biofluids [276,277]. Analytes in sweat and selected detection methods were summarized by Bariya et al. [261] in a review that addressed the state-of-the-art wearable sweat sensors within common athletic accessories designed as wristbands or headbands or medically dedicated patch-like devices. The medical devices allowed continuous and comfortable fitness monitoring due to their great flexibility for minimal obstruction of motions or discreet adherence to the skin. It was concluded that the flexible and wearable devices could allow data collection and integration for medical purposes, even for prognosis or prediction of illness before the onset of symptoms [278]. In this direction, Imani et al. [279] introduced a skin-worn hybrid sensing system for comprehensive fitness monitoring. The system simultaneously real-time monitored biochemicals (lactate) and electrophysiological signals (electrocardiogram). The three-electrode amperometric lactate biosensor and a bipolar electrocardiogram sensor were incorporated onto a flexible substrate and mounted on the skin. The studies on human subjects revealed negligible crosstalk between physiochemistry and electrophysiology sensors during simultaneous activation of the two sensing modalities. The results concluded that the new technology is a new class of hybrid sensing devices. Furthermore, recent developments combined microtechnologies on the same device. For instance, one platform incorporated MN to measure temperature, humidity, glucose, and pH sensors in situ and wirelessly transmit the results for monitoring and adequate therapy. The stretchable system consisted of (1) an electrochemical interface made of gold mesh and gold-doped graphene for a constant transfer of electrical signals and (2) polymeric MN thermally activated for transcutaneous delivery of Metformin. Therefore, the future is of wearable epidermal biosensors for real-time analysis of biomarkers in sweat or interstitial fluid. 

### 5.5. Wearable Microfluidic Devices 

Skin-interfaced wearable sensors for sweat analysis, based on integrated microfluidic channels coupled with colorimetric or electrochemical sensors, demonstrated promising prospects for application in real-time, non-invasive athletic monitoring and personalized clinical medicine. The latest advances in skin-interfaced microfluidics for noninvasive sweat sensing were discussed in detail in recent review articles [280,281]. Skin-mounted microfluidic devices are designed to collect, store and chemically analyze the sweat released by the eccrine glands, consisting of a microfluidic device, sensing system, and electronic components. These modern noninvasive healthcare systems can acquire, with high precision, different physiological information such as sweat loss, information regarding metabolites, and electrolyte balance. 

These devices should be biocompatible, maintain a conformal contact with the skin for optimal sweat collection and quickly transport the sweat to the sensing area, avoiding the loss by evaporation or dilution. Regarding materials, the microfluidic system is usually made of biocompatible, soft silicone elastomers with a low Young’s modulus. Usually, PDMS is constructed using a soft lithography technique and biocompatible materials like hypoallergenic silicone or medical-grade acrylic were selected to construct the adhesive layer.

The first generation of microfluidic systems used in sweat analysis [269] consisted of simple networks of microfluidic channels and reservoirs. However, these microchannels recently evolved, having a more complex design so that the capture and transportation of sweat from the eccrine glands to the sensing area were programmed.

In 2016, Koh et al. fabricated a microfluidic system mounted on the skin with microchannels and reservoirs so that perspiration circulated spontaneously through it due to the capillary effect [269]. The sweat was conducted to the sensing area and was colorimetrically tested for chloride and hydronium ions, glucose, and lactate. Choi et al. in [268] proposed high performance soft, skin-mounted microfluidic networks with bursting capillary valves, which could follow the temporal variation of electrolyte balance and biomarker concentration. Nyein et al. in [282] investigated the composition of sweat from different parts of the body using patches with microfluidic chips and sensing electrodes. They were able to predict the total body fluid and electrolyte losses during exercise, and correlate sweat glucose level with its concentration in blood.

## 6. Conclusions and Perspectives

Since active monitoring of health conditions is a pressing and continuous priority, efforts have focused on developing methods and devices to support the diagnostic, therapeutic and preventive approaches. The recent development of technology has opened new avenues for effective and patient-friendly measurement of physical and biochemical physiological parameters and even for ongoing analysis for rapid and personalized interventions. Either minute collection of biofluids for biomarkers detection or the collection of large sets of data on physical and chemical homeostatic parameters could capture meaningful and timely health status variations to enable prompt interventions and to allow appropriate prevention. Therefore, over the past few years, efforts have consolidated the progress in designing and manufacturing e-skin systems for cost-efficient products. A few aspects have consolidated the profile of e-skin: the quality materials used with adequate flexibility [284,285], stretchability [286], transparency [287]), light weightiness [28,288], high precision sensory functions [4], and the sensitive techniques for complex molecules detection. 

Studies have established the feasibility of the concept of using organic transistors for the active-matrix backplane of e-skin. However, the low carrier mobility of organic transistors imposed the inorganic crystalline semiconductors with miniaturized dimensions and superior mechanical flexibility [284,289]. There have been newly employed materials which have contributed to various kinds of flexible and stretchable devices based on an ultrathin [290] and stretchable design [28,291]. The developed systems were dedicated to monitoring individual health status and delivering the corresponding feedback therapy [292,293]. However, the new designs required improved scalability and multifunctionality to comply with the desired forms of the physical and chemical sensing e-skin and future medical applications. One more aspect considered when developing e-skin was the previous biosensors’ limitations caused by the single-analyte monitoring capacity and the lack of on-site signal processing circuitry and sensor calibration mechanisms [294]. Therefore, further development of flexible platforms that can house several sensors simultaneously is a priority. The fully integrated platforms for continuous and simultaneous detection of several physiological parameters built inside passively activated microfluidic systems and from biocompatible protective compartment-sealing membranes may also be coupled with capabilities for wireless data collection. Such an extended capability will contribute to the e-skin potential as real-time, multi-parameter concurrent analysis with little or no discomfort to users. This solution overcame the problems associated with blood sample collection and provided higher compliance among various users (e.g., athletes, patients). 

The improved features will further develop the diagnostic application of e-skin based on the recent focus collecting body biomarkers to detect the presence of different biomarkers in the body and include their measurement for physiological parameters, biomolecules, and bodily fluids monitoring. Furthermore, the progress of personalized medicine- and IoT-related fields is the motor for improved and consistent manufacturing of reliable and stable systems that integrate more sensing modalities [295]. Therefore, the following steps will employ artificial intelligence to address the motion artefacts, the interaction of e-skin with the human skin for a level of comfort and accurate feedback. Moreover, it has been acknowledged that the intrinsic complexities of using antibodies or aptamers as biorecognition elements have caused difficulties when developing specific and multimodal sensors. In this case, achieving technological standards could contribute tremendously towards a better molecular diagnostic and personalized therapy. The complexity of the multidisciplinary work in perfecting the e-skin will also strengthen the collaboration between academia and industry to achieve long-term stability of the integrated e-skin platforms capable of facilitating ongoing monitoring and timely diagnostics for efficient therapy and prevention. The evolving technology will consolidate the transition from conventional electronics and open new avenues for multifunctional, smart, user-friendly, and cost-efficient products. The variety of considerations presented have established challenges and opportunities for a new era of sensor technology to enable noninvasive investigation at molecular levels for personalized and predictive healthcare. The crucial goal of flexible and wearable health-monitoring devices for periodic health monitoring is to allow data to be collected and integrated for medical purposes and even to predict illness before the onset of symptoms.
